# Impaired fibrinolysis and lower levels of plasma α_2_-macroglobulin are associated with an increased risk of severe asthma exacerbations

**DOI:** 10.1038/s41598-017-11467-8

**Published:** 2017-09-08

**Authors:** Stanislawa Bazan-Socha, Lucyna Mastalerz, Agnieszka Cybulska, Lech Zareba, Romy Kremers, Michal Zabczyk, Grazyna Pulka, Teresa Iwaniec, Jan G. Bazan, Coenraad Hemker, Anetta Undas

**Affiliations:** 1 Department of Internal Medicine, Jagiellonian University Medical College, Krakow, Poland; 2 Faculty of Mathematics and Natural Sciences, University of Rzeszow, Rzeszow, Poland; 3 Synapse Research Institute, Cardiovascular Research Institute Maastricht, Maastricht University, Maastricht, The Netherlands; 4 Centre for Medical Research and Technologies, The John Paul II Hospital, Krakow, Poland; 5 University Hospital, Allergy and Clinical Immunology Department, Krakow, Poland; 6 Interdisciplinary Centre for Computational Modelling, University of Rzeszow, Rzeszow, Poland; 7 Institute of Cardiology, Jagiellonian University Medical College, Krakow, Poland

## Abstract

Recently we have reported that asthma is associated with enhanced plasma thrombin formation, impaired fibrinolysis and platelet activation. In the present study we investigated whether described prothrombotic blood alterations might predispose to thromboembolic events or asthma exacerbations. In 164 adult asthmatics we assessed clinical events during 3-year follow-up and analyzed their associations with measured at baseline prothrombotic blood parameters. Data were obtained from 157 (95.7%) of the asthma patients. We documented 198 severe asthma exacerbations (64/year), which occurred in 53 subjects (34%). These patients were older (p = 0.004), had worse asthma control (p = 0.02) and lower spirometry values (p = 0.01), at baseline. Interestingly, this subgroup had longer clot lysis time (CLT), as well as lower α_2_-macroglobulin (p = 0.038 and p = 0.04, respectively, after adjustment for potential confounders). Increased CLT and lower α_2_-macroglobulin were demonstrated as independent predictors of asthma exacerbation in multiple regression model. Moreover, we documented two episodes of deep vein thrombosis (1.3%), and eight acute coronary syndromes (5.1%). Patients who experienced thromboembolic events (n = 10, 6.4%, 2.1%/year) had lower α_2_-macroglobulin (p = 0.04), without differences in efficiency of fibrinolysis and thrombin generation. Impaired fibrinolysis and lower levels of α_2_-macroglobulin might predispose to a higher rate of asthma exacerbations, suggesting new links between disturbed hemostasis and asthma.

## Introduction

Asthma is a highly prevalent, chronic respiratory disease characterized by wheezes, shortness of breath, chest tightness and/or cough, along with the variable airflow airway limitation^1^. Both symptoms and bronchial obstruction may vary over time and are often triggered by factors, such as exercise, allergen or irritant exposure, change in weather, or viral respiratory infection. Symptoms and airflow limitation may resolve spontaneously or in response to medication, but sometimes asthma subjects can experience exacerbations that may be severe or even life-threatening^1, 2^. These conditions are distressing to patients and result in considerable utilization of health care resources and loss of work productivity or school attendance^3^. Having had at least one exacerbation is an important risk factor for recurrent exacerbations suggesting an “exacerbation-prone” subset of asthmatics. Factors underlying the “exacerbation-prone” phenotype are incompletely understood. They include extrinsic factors: cigarette smoking, medication noncompliance, psychosocial factors, and co-morbidities such as gastroesophageal reflux disease (GERD), rhinosinusitis, obesity, and intolerance to non-steroidal anti-inflammatory medications, along with intrinsic factors e.g. deficient epithelial cell production of the anti-viral interferons^3^.

Asthma is related to chronic airway inflammation, which persists even when symptoms are absent. Inflammation and coagulation interact with each other in a number of physiological and pathological conditions. There is growing evidence for heightened activation of blood coagulation in the airways of asthmatic subjects and for pro-coagulant plasma protein leakage into the bronchoalveolar space^4^. However, it is unknown whether this local phenomenon is associated with systemic blood coagulation, severity of airway inflammation, or asthma exacerbation rate. A molecular link between coagulation and inflammation might be explained by specific G-protein-coupled protease activated receptors (PARs), which are expressed on various airway cells, including epithelial and smooth muscle cells, as well as inflammatory infiltrating cells^5^. Activation of these receptors, e.g. by thrombin, factor (F)Xa and complex of tissue factor/FVIIa, leads to the overproduction of inflammatory cytokines, including interleukin(IL)-8, IL-6, P-selectin, and platelet derived growth factor amplifying inflammation and contributing to the asthma exacerbation^5–7^. PARs may also participate in the airway remodelling, another important feature of asthma that compromises asthma control^6, 7^. Prothrombotic haemostatic imbalance in asthma has been postulated after a rising number of reports on the increased risk of thromboembolic events in subjects with this disease^8–11^. Recently we demonstrated that persistent asthma is accompanied by enhanced thrombin generation, impaired fibrinolysis and platelet activation in circulating blood^12^. Similar observations have been documented by Sneeboer *et al*.^13^, who also showed increased thrombin formation, together with higher levels of plasminogen activator inhibitor -1 (PAI-1), D-dimer, von Willebrand factor, and plasmin-α_2_-antiplasmin complexes in this disease. Moreover, plasma clot studies performed by Tomasiak-Lozowska *et al* ^14^. indicated reduced fibrinolytic capacity in asthmatics. However, clinical implications of the prothrombotic state in asthma remain to be established.

In the present study we sought to investigate whether prothrombotic alterations in circulating blood of asthmatic subjects, demonstrated by us previously^12^, might be associated with a risk of severe exacerbations or thromboembolic events in these patients during follow-up.

## Results

### Patient characteristics

Follow-up data is obtained from 157 asthmatics (95.7%). Seven patients (4.3%) were lost to follow-up. Clinical characteristics of asthma and control subjects were described in our previous article in detail^12^. Of note, both studied groups were well matched, except for GERD, which was more common in asthma subjects (36 [23%] vs. 5 [7%], p < 0.0001). Among asthmatics at baseline 20 (13%) subjects were diagnosed with sporadic asthma, 33 (21%) with persistent mild, 45 (29%) with moderate and 59 (38%) were severe asthmatics. Atopy was observed in 88 (56%) asthma patients, including 13 with sporadic asthma, 16 with mild, 24 with moderate and 35 severe asthmatics, representing 65%, 48%, 53% and 59% of all patients from these 4 asthma subsets, respectively.

All persistent asthmatics (n = 138, 88%) received inhaled corticosteroids, 119 (76%) long-acting β_2_-agonists, 36 (23%) montelukast, 24 (15%) theophyllin, and 32 (21%) were on oral corticosteroids. All asthmatics on theophyllin and all but one on montelukast received inhaled corticosteroids. Nine patients on montelukast (25%) and 13 on theophyllin (54%) were also treated with oral corticosteroids.

### Laboratory variables

Results of laboratory tests performed at baseline in asthmatics and controls have been presented previously in detail^12, 15^. Briefly, asthmatics were characterized by elevated inflammatory markers, including high-sensitivity C-reactive protein (hsCRP) (1.24 [1.01–1.47] vs. 0.87 [0.6–1.13] mg/l, p = 0.03), IL-6 (4.57 [4.41–4.73] vs. 3.06 [2.56–4.1] pg/ml, p < 0.0001), tumor necrosis factor α (TNFα) (3.95 [3.82–4.08] vs. 2.91 [2.26–3.7] pg/ml, p < 0.0001), and fibrinogen (3.55 [3.47–3.63] vs. 3.36 [3.3–3.4] g/l, p = 0.001). They had also unfavourable altered plasma thrombin formation expressed as markedly higher endogenous thrombin potential (ETP) (1506 [1481–1531] vs. 1255 [1221–1287] nmol/l thrombin x min, increased thrombin peak (283.6 [277.2–289.9] vs. 200.5 [192–208.9] nmol/l), and faster rate of thrombin formation (time to thrombin peak) (5 [4.88–5.12] vs. 5.92 [5.69–6.14] min) (all, p < 0.0001). In asthma we also documented longer clot lysis time (CLT) (95 [89.9–100] vs. 83.2 [80.5–85.87] min, p = 0.002), which reflected impaired plasma fibrinolytic capacity. Moreover, asthma subjects had higher platelet factor 4 (PF4) (146.2 [144–148.3] vs. 97 [94.28–99.72] ng/ml, p < 0.0001), as well as raised α_2_-macroglobulin levels (15.07 [14.43–15.71] vs. 12.7 [12.1–13.3] nmol/l, p = 0.0002). Of note, prothrombotic blood alterations were related to the low-grade IL-6-mediated inflammatory state in this disease^15^.

### Clinical outcomes in follow-up

Among 157 asthmatics, whose data were available during follow-up, one patient died due to brain glioma 34 months after enrolment. There was no asthma-related mortality, but one 64-year-old woman had cardiac arrest in a severe asthma exacerbation, followed by a successful resuscitation.

#### Thromboembolic events

None of the patients suffered from a stroke or transient ischemic attack (TIA).

Eight asthmatics (5 women and 3 men, 5.1%) were diagnosed with acute coronary syndrome (ACS). These subjects were older (63.5 [59.2–67.8], vs. 53 [51.5–54.5] years, p < 0.0001), often previously diagnosed with coronary heart disease (CHD) (n = 6, 3.8%, p < 0.0001) and longer suffered from asthma (27.5 [23.5–31.5] vs. 11 [9.7–12.3] years, p < 0.0001), than remaining asthmatics. No pulmonary embolism was recorded, but two women with asthma (1.3%), 49- and 55-year-old, were diagnosed with deep vein thrombosis (DVT), 6 and 10 months after enrolment. Surprisingly, among all 49 asthmatic women aged 45–60, these with DVT had the two lowest concentrations of α_2_-macroglobulin (7.23 and 9.46 nmol/l, median in this group was 15.06 [13.9–16.2] nmol/l), and the two highest values of ratios of ETP to α_2_-macroglobulin (171.1 and 197.2, median in this group was 105 (98.7–111.3) and peak thrombin to α_2_-macroglobulin (36.1 and 39.7, median in this group was 17.05 [15.69–18.41]).

When patients with arterial or venous thromboembolism (n = 10, 6.4%, 2.1/year) were analyzed collectively, this subset was also older (62.5 [58.5–66.5] vs. 53[51.5–54.5] years, p = 0.02), longer suffered from asthma (27.5 [23–32] vs. 11 [9.7–12.4] years, p = 0.02), and often was previously diagnosed with CHD (n = 6, 3.8%, p < 0.0001). These subjects were also characterized by lower α_2_-macroglobulin (12 [10.4–13.4] vs. 15[14.31–15.6] nmol/l, p = 0.04) and thrombin-α_2_-macroglobulin complex formation (11.9 [10.4–13.4] vs. 15.06 [14.4–15.7] nmol/l, p = 0.04).

#### Severe asthma exacerbations

We documented 198 (64/year) severe asthma exacerbations, which occurred in 53 (34%) patients. Among them 13 (25%) asthmatics had a single exacerbation, whilst 33 (62%) had at least three such complications. In subjects who had at least one exacerbation, 3 (6%) patients were previously diagnosed with sporadic asthma, 4 (8%) with persistent mild, 16 (30%) with moderate and 30 (57%) were severe asthmatics.

Clinical and laboratory characteristics of the asthma subjects with and without severe exacerbations are given in Tables 1 and 2. Asthmatics with documented exacerbations had the same male to female ratio and age of asthma onset, but they were older (56 [53.6–58.4] vs. 51 [49.2–53] years, p = 0.004), longer suffered from asthma (16 [13.6–18.4] vs. 10 [8.5–11.5] years, p = 0.006), as well as had worse asthma control, expressed as the Asthma Control Test score (19 [18.3–19.7] vs. 21 [20.5–21.5], p = 0.02), at baseline. Moreover, these subjects were characterized by a higher frequency of hypertension and GERD, as well as lower values of forced expiratory volume in 1 second (FEV_1_), also after correction for sex, age, and body mass index (BMI) (p = 0.01).

**Table 1:** Baseline demographic and clinical characteristics of the asthma subjects both those, who did not experience severe asthma exacerbation or those with at least one exacerbation during 3-year follow-up.

	asthmatics without exacerbation, n = 103	asthmatics with at least one exacerbation, n = 53	p-value
Age, years	51 (49.2–53)	56 (53.6–58.4)	0.004
Age of asthma onset, years	33 (30.9–35.1)	33 (30.1–35.9)	0.63
Male gender, n (%)	29 (28)	12 (23)	0.58
Body mass index, kg/m^2^	26.33(25.79–26.87)	26.56 (25.8–27.4)	0.13
Current smoking, n (%)	12 (11.6)	4 (7.5)	0.6
Number of pack-years	13 (9.8–16.2)	13.5 (7.3–19.7)	0.7
Atopy, n (%)	60 (58)	28 (53)	0.63
Arterial hypertension, n (%)	26 (25)	27 (51)	0.002
Diabetes mellitus, n (%)	9 (9)	5 (9)	0.88
Hypercholesterolemia, n (%)	48 (47)	34 (64)	0.06
Coronary heart disease, n (%)	5 (5)	8 (15)	0.06
Gastroesophageal reflux disease, n (%)	11 (11)	25 (47)	<0.0001
Medications used:			
Antihypertensives, n (%)	24 (23)	27 (51)	0.0009
Statins, n (%)	12 (12)	10 (19)	0.33
Aspirin, n (%)	3 (3)	5 (9)	0.17
Oral estrogens, n (%)	14 (14)	3 (6)	0.21
Asthma duration, years	10 (8.5–11.5)	16 (13.6–18.4)	0.006
Asthma Control Test, score	21 (20.5–21.5)	19 (18.3–19.7)	0.02
Asthma severity (GINA)			
intermittent, n (%)	17 (17)	3 (6)	< 0.0001
persistent mild, n (%)	29 (28)	4 (8)	< 0.0001
persistent moderate, n (%)	28 (27)	16 (30)	0.02
persistent severe, n (%)	29 (28)	30 (57)	1
Asthma treatment:			
Inhaled corticosteroids, n (%)	90 (87)	49 (92)	0.49
Long-acting β_2_-agonists, n (%)	71 (69)	47 (89)	0.01
Montelukast, n (%)	23 (22)	13 (25)	0.91
Theophyllin, n (%)	12 (12)	12 (23)	0.12
Oral corticosteroids, n (%)	11 (11)	21 (40)	0.0001
FEV_1_, % of predicted value	91 (87.45–94.55)	85 (81.4–88.6)	0.007
FEV_1_/VC	0.77 (0.76–0.78)	0.76 (0.74–0.78)	0.1

Categorical variables are presented as numbers (percentages), continuous variables as a median with a 95% confidence interval. Continuous variables were compared by the Mann-Whitney U-test, while categorical variables by χ^2^ test. Abbreviations: FEV_1_ - Forced expiratory volume in 1 second, FEV_1_/VC - Forced expiratory volume in 1 second/vital capacity, GINA - Global Initiative for Asthma, n – number.

**Table 2:** Selected laboratory variables measured at baseline in the asthma subjects both those, who did not experience severe asthma exacerbation or those with at least one exacerbation during 3-year follow-up.

	asthmatics without exacerbation, n = 103	asthmatics with at least one exacerbation, n = 53	p-value
**Laboratory tests**			
Blood platelets, 10^3^/μl	214 (208–220)	217 (206–227)	0.18
Fibrinogen, g/l	3.5 (3.41–3.59)	3.6 (3.45–3.75)	0.22
Prothrombin, %	107 (105.4–108.6)	109 (106.8–111)	0.56
high-sensitivity C-reactive protein, mg/l	1.06 (0.78–1.33)	1.78 (1.35–2.21)	0.06
Immunoglobulin E, log_10_, IU/ml	1.96 (1.88–2.04)	1.83 (1.75–1.91)	0.16
Blood eosinophilia/μl	160 (137–183)	170 (138–202)	0.65
Interleukin 6, pg/ml	4.51 (4.32–4.7)	4.81 (4.52–5.1)	0.18
Tumor necrosis factor α, pg/ml	3.95 (3.79–4.11)	4.02 (3.79–4.25)	0.75
Platelet factor 4, ng/ml	148.1 (145.4–150.9)	142.8 (139.3–146.3)	0.46
**α**_**2**_ **-macroglobulin**			
α_2_-macroglobulin, nmol/l	15.9 (15.1–16.7)	13.2 (12.2–14.2)	0.03
**Thrombin generation and computational assessment of thrombin dynamics**			
Thrombin peak, nmol/l	282.9 (275.5–290.3)	282.5 (270.5–294.5)	0.53
Endogenous thrombin potential, nmol/l thrombin x min	1504 (1474–1535)	1509 (1463–1556)	0.86
Total amount of prothrombin converted, nmol/l	918.8 (900–937.4)	887.3 (858.5–916)	0.36
Thrombin-antithrombin complex formation, nmol/l	856.2 (838.1–874.3)	833.2 (805.6–860.5)	0.49
Thrombin-α_2_-macroglobulin complex formation, nmol/l	15.9 (15.13–16.67)	13.2 (12.24–14.16)	0.02
**Fibrinolysis**			
Clot lysis time, min	90.3 (83.85–96.75)	106.8 (98.9–114.7)	0.006
Plasminogen, %	105 (102.8–107.2)	107 (104.3–109.8)	0.96
Plasminogen activator inhibitor-1, ng/ml	30 (28.12–31.88)	29.6 (26.6–32.6)	0.26

For legend and abbreviations see Table 1.

**Figure 1:**
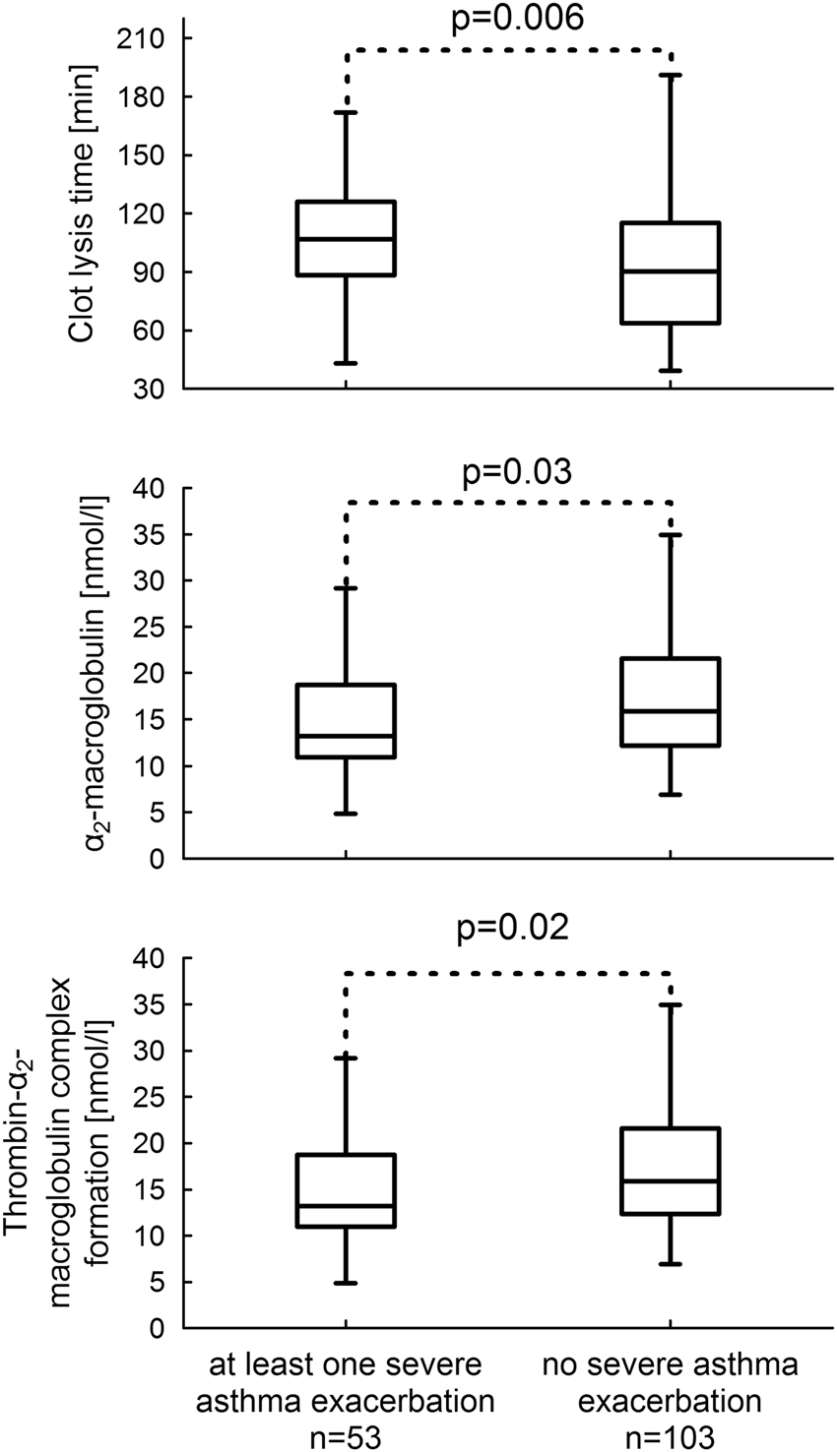
Laboratory variables that differentiated asthmatics with at least one severe exacerbation and those, who did not experience severe asthma exacerbation during the whole 3-year period of follow-up. Data is presented as median, interquartile range, and minimum and maximum value.

Among laboratory parameters describing prothrombotic plasma properties, asthmatics with at least one exacerbation were characterized by longer CLT (106.8 [98.9–114.7] vs. 90.3 [83.85–96.75] min, p = 0.006) (Fig. 1) and lower levels of α_2_-macroglobulin (13.2 (12.2–14.2) vs. 15.9 (15.1–16.7), nmol/l p = 0.03) (Fig. 1), also after correction for age, age of asthma onset, sex, BMI, asthma severity, according to GINA (or FEV_1_), oral corticosteroids used at baseline, and co-morbidities, i.e. arterial hypertension and GERD (F = 4.24, p = 0.038; and F = 4.18, p = 0.04, respectively).

Higher CLT (109.3 [100.8–117.8] vs. 86.8 [77.8 vs. 95.9] min, p = 0.002) and lower α_2_-macroglobulin (13.3 [12.26–14.4] vs. 15.8 [14.9–16.9] nmol/l, p = 0.04) were also documented in subjects with at least one asthma exacerbation, when analysis was limited to the moderate and severe asthmatics (n = 46 vs. without exacerbation, n = 57).

Both these laboratory variables were also shown as independent predictors of asthma exacerbation in a multiple logistic regression model (Table 3).

**Table 3:** A multiple logistic regression model assessing independent risk of asthma exacerbation.

	Evaluation factor (95% CI)		Standard error	Wald’s statistic	p-value	Odds ratio (95% CI)
Asthma duration, years	0.037 (0.011 to 0.063)		0.013	7.536	0.006	1.037 (1.011–1.065)
Asthma Control Test, number of scores	−0.05 (−0.1 to −0.001)		0.025	3.991	0.046	0.951 (0.905–0.999)
Clot Lysis Time, min	0.007 (0.0001 to 0.013)		0.003	3.862	0.049	1.007 (1.001–1.013)
α_2_-macroglobulin, nmol/l	−0.064 (−0.121 to −0.008)		0.029	5.041	0.025	0.938 (0.886–0.992)
Adjustment statistics						
		Test’s statistic		p-value		
Hosmer-Lemeshof test		7.33		0.5		

Abbreviations: CI - confidence interval In this model the resulting standardized coefficient of regression (with 95% confidence intervals [95%CIs]) assesses probability (risk) of asthma exacerbation, when that particular independent variable increases/decreases by 1 unit and other variables in the model (listed in column 1) are unchanged.

Analysis of thrombin generation kinetics showed that the only parameter, that was associated with increased risk of exacerbation, was thrombin-α_2_-macroglobulin complex formation, closely related to α_2_-macroglobulin levels, being lower in this group of asthmatics (13.2 [12.24–14.16] vs. 15.9 [15.13–16.67] nmol/l, p = 0.02) (Fig. 1), also after adjustment for potential confounders (F = 4.48, p = 0.036).

**Figure 2:**
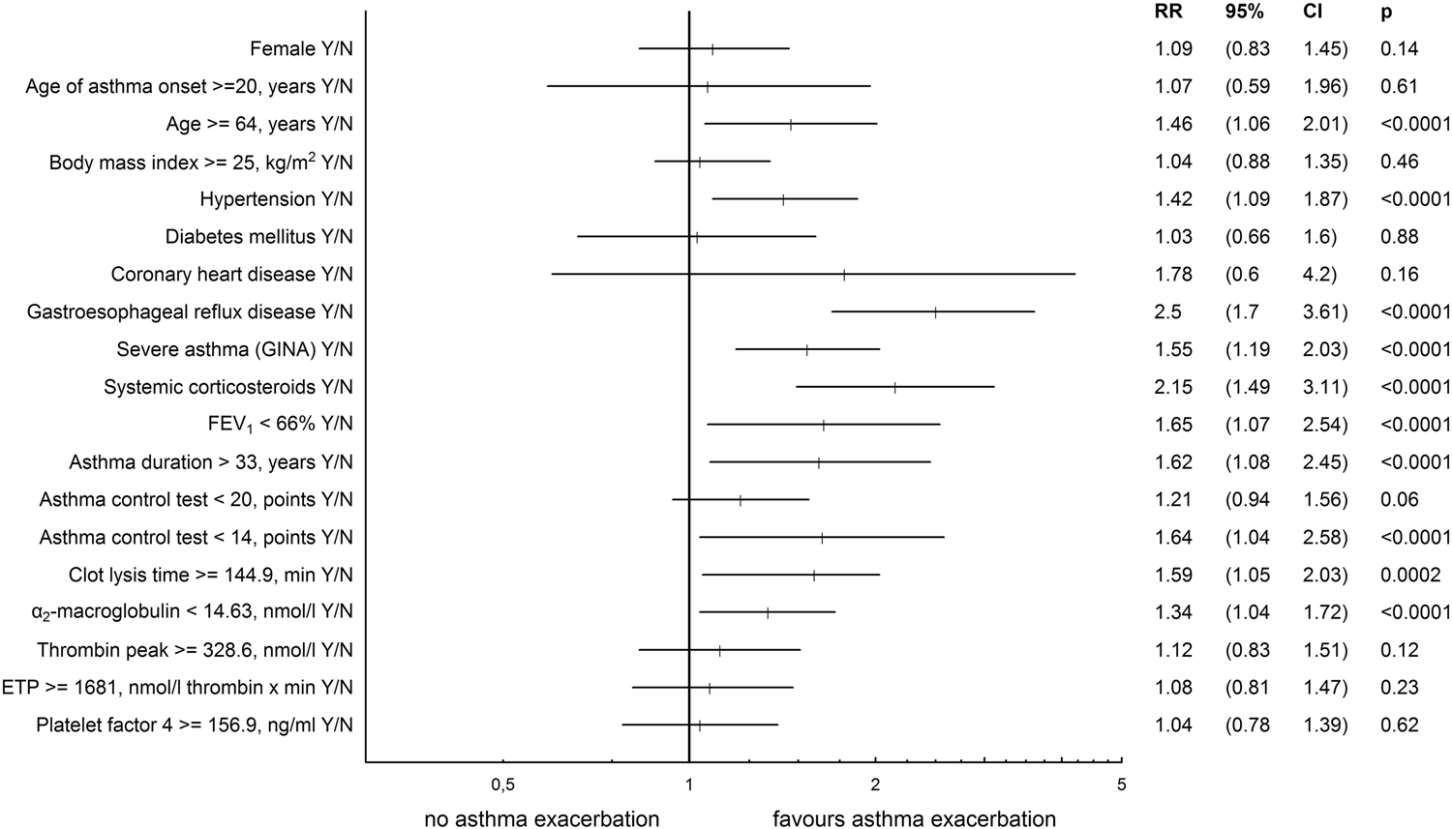
Impact of selected clinical and laboratory variables on the risk of development of at least one severe asthma exacerbation, expressed as relative risk ratios (RRs) with 95% confidence intervals (CIs). As expected, higher RR of asthma exacerbation was demonstrated in subjects who were older and longer suffered from asthma, had hypertension and gastroesophageal reflux disease, as well as in those with more severe type of disease, particularly on oral corticosteroids at baseline, and with lower values of forced expiratory volume in 1 second (FEV_1_). Moreover, asthma subjects with Clot Lysis Time ≥144.9 min and α_2_-macroglobulin <14.63 nmol/l had higher RRs of asthma exacerbation than remaining patients (numerical values were calculated based on ROC curves). Abbreviations: RR - relative risk ratio, 95%CI − 95% confidence interval, GINA - Global Initiative for Asthma, FEV_1_ - forced expiratory volume in 1 second, ETP - Endogenous Thrombin Potential: parameter describing thrombin generation capacity.

Figure 2 demonstrates the impact of selected clinical and laboratory variables on the risk of asthma exacerbation, expressed as relative risks (RRs). As expected, the most important parameters describing risk of asthma exacerbation were severe asthma (RR 1.55 [95%CI: 1.19–2.03), p < 0.0001], treatment with oral corticosteroids at baseline (RR 2.15 [95%CI: 1.49–3.11], p < 0.0001), and GERD (2.5 [95%CI: 1.7–3.61], p < 0.0001). Moreover, asthmatics characterized by CLT ≥ 144.9 min and α_2_-macroglobulin <14.63 nmol/l had higher risks of asthma exacerbation than the remaining patients (RR 1.59 [95% CI: 1.05–2.03], p = 0.0002 and RR 1.34 [95% CI: 1.04–1.72], p < 0.0001, respectively) (Fig. 2).

**Figure 3:**
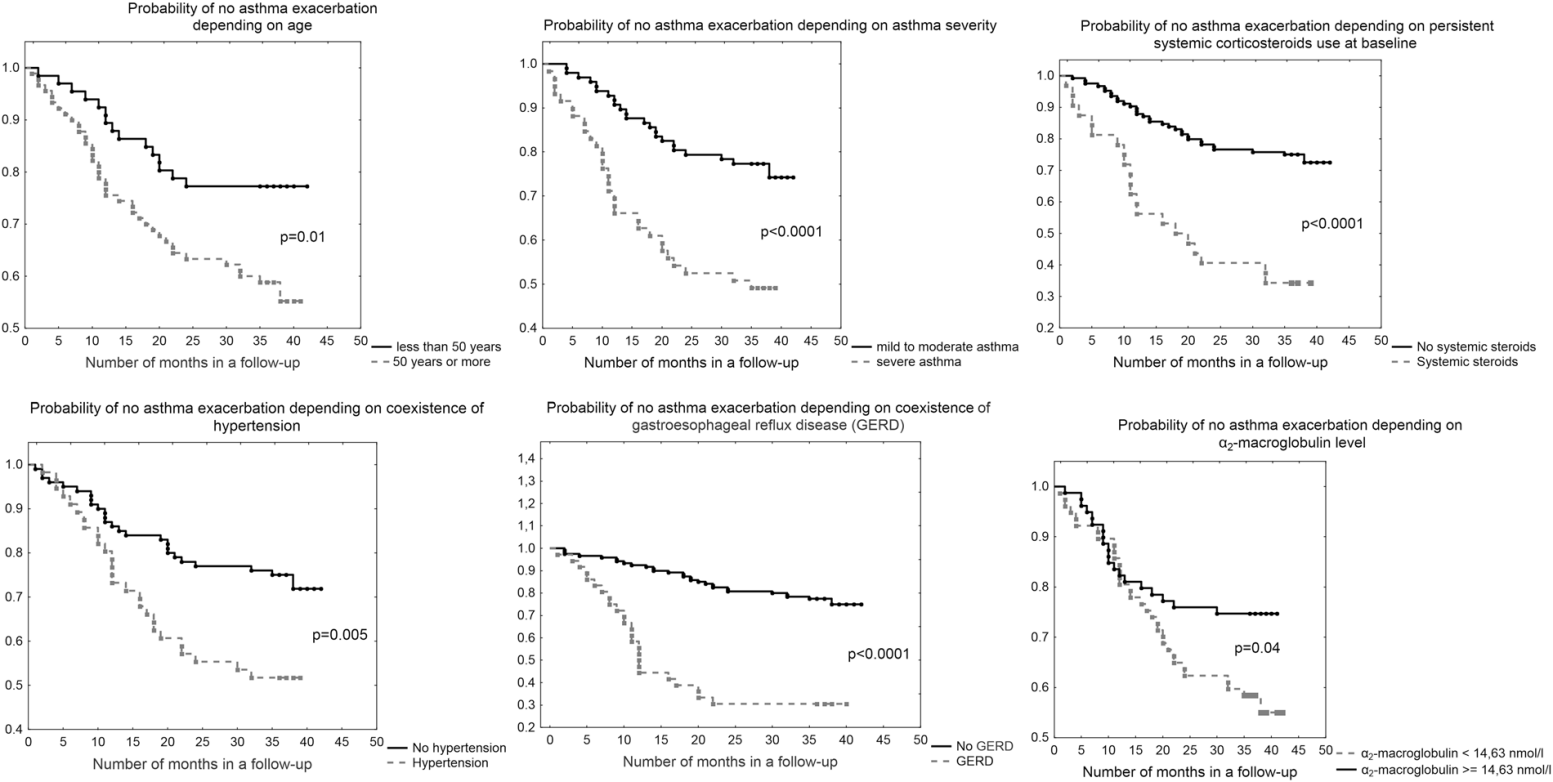
Kaplan-Meier plots in estimation of asthma exacerbation rate in the time. Faster exacerbations were demonstrated in subjects 50 years old or more, with severe asthma, particularly on oral corticosteroids at baseline, as well as with hypertension and gastroesophageal reflux disease as co-morbidities, and with α_2_-macroglobulin <14.63 nmol/l.

Among subjects, who were exacerbated, 14 (26%) developed this complication for the first time during the first 8 months of follow-up and 29 (55%) in the first year. Only 5 asthmatics (9%) had a first exacerbation after 24 months following blood collection. The Kaplan-Meyer plots revealed that patients aged 50 years or more (p = 0.01), with severe asthma, particularly on oral corticosteroids at baseline (both, p < 0.0001), with arterial hypertension (p = 0.005) and GERD (p < 0.0001), as well as those with α_2_-macroglobulin <14.63 nmol/l (p = 0.04), had higher risk of faster occurrence of the first asthma exacerbation after enrolment (Fig. 3). A Cox proportional hazards model also demonstrated that lower α_2_-maroglobulin (β = −0.048 [95% CI: −0.096 to −0.00003], p = 0.04), together with lower thrombin-α_2_-macroglobulin complex formation (β = − 0.05 [95% CI: −0.098 to −0.0015], p = 0.035) and lower FEV_1_ (β = −0.02 [95% CI: −0.04 to −0.008], p = 0.001), were predictors of a faster first exacerbation in the time. We were not able, however, to demonstrate any biomarker that can predict the risk of a second or a total number of asthma exacerbations during follow-up.

## Discussion

In the present study we show for the first time that impaired fibrinolysis and lower levels of α_2_-macroglobulin may contribute to the increased risk of severe asthma exacerbation. This unexpected finding suggests new links of disturbed hemostasis and asthma. There are many known independent risk factors of asthma worsening, including cigarette smoking, allergen exposure if sensitized, uncontrolled asthma, low FEV_1_, sputum and blood eosinophilia, and co-morbidities such as GERD, obesity, and food allergy^1, 3^. In the current study we have shown that also prothrombotic alterations of circulating blood may contribute to the increased risk of asthma exacerbation, particularly if associated with lower α_2_-macroglobulin level.

Thrombin generation determined in the CAT assay is a reproducible and reliable automated tool for assessment of thrombotic-haemostatic functions of blood in an integrated manner^16–18^. Previously we have demonstrated that asthmatics are characterized by increased thrombin formation, which allowed us to report a prothrombotic state and speculated that it might be associated with increased risk of thromboembolic complications^12^. The present study neither excludes nor affirms this concept because of a low number of subjects with thrombotic events in the present asthma cohort during a relatively short-term follow-up. Regarding asthma exacerbations, there was no association between parameters of thrombin generation and their risk in this study. An association, however, was demonstrated for longer CLT, which indicates impaired global plasma fibrinolytic capacity. CLT measured using a plasma-based assay has been successfully used to determine the efficiency of fibrinolysis in various diseases including subjects at risk of thrombosis^19–22^. Previously we demonstrated that one of the most important determinants of longer CLT among asthmatics was higher ETP^12^. This observation confirmed that more compact fibrin clot networks typically formed in the presence of higher thrombin amounts are lysed slowly in asthma, similarly to the patients at risk of venous thromboembolic diseases^21^. Moreover, it also suggests that increased thrombin generation might at least indirectly predispose to the increased rate of exacerbations in asthma.

In the present study we also demonstrated that the α_2_-macroglobulin may participate in the pathology of asthma exacerbation. In a logistic regression model low α_2_-macroglobulin concentrations appear as an independent predictor of severe asthma exacerbation. This observation allows us to speculate that higher α_2_-macroglobulin in asthma subjects has a protective role. α_2_-macroglobulin acts as a universal protease inhibitor, capable of binding various host or foreign peptides and particles, cytokines, and growth factors, plasmin and thrombin, as well as kallikrein^23, 24^. In our previous study^12^ we hypothesized that circulating levels of α_2_-macroglobulin, increased in asthmatics, could be raised in response to activated blood coagulation, contributing to the attenuation of a prothrombotic state^25^. The present results support this hypothesis, since plasma levels of α_2_-macroglobulin were lower in subjects with thromboembolic events. Moreover, based on these results we may speculate that α_2_-macroglobulin has also a beneficial role in the regulation of inflammatory response in asthma, protecting against exacerbation of the disease. The relative deficiency of α_2_-macroglobulin in circulating blood of asthmatic patients may lead to the higher levels of pro-inflammatory and prothrombotic agents, which affect systemic inflammation and asthma worsening^4, 5^. However, the recent report from COPDGene and SPIROMICS investigators published by Keene *et al*.^26^ demonstrated that in subjects with chronic obstructive pulmonary disease (COPD), among 90 blood biomarkers tested at baseline, only two, including α_2_-macroglobulin, had a positive predictive value for future severe exacerbations. COPD similarly to asthma is obstructive by nature, but its pathophysiology is different, which might explain a different role of this protein in the two diseases. Undoubtedly, those and our results clearly indicate that α_2_-macroglobulin is involved in obstructive lung disorders. Further studies are needed to elucidate the mechanism underlying this involvement.

It should be noted that animal studies in experimental settings provide abundant evidence for beneficial effects of interventions with variable anticoagulants and fibrinolytic medications on disturbed pulmonary hemostatic balance, leading to the reduction in the airway inflammation^4^. Obviously, anticoagulant therapy is not used in asthma treatment nowadays, however, favourable effects of inhaled heparin observed in humans on airway inflammation and asthma symptoms might be partially related to the suppression of excessively activated thrombin formation, as well as elimination of harmful thrombin-mediated cellular inflammatory effects, e.g. via PAR receptors^5^.

### Study limitations

The group of patients was relatively small. For this reason the study was underpowered to analyse thromboembolic events, which is an important bias in regards of the studied biomarkers. However, the present study was sufficiently powered to demonstrate relations of clinical and laboratory variables to severe asthma exacerbations. We determined each variable at a single time point before follow-up, and therefore we cannot exclude changes of the variables studied over time, particularly after first asthma exacerbation. We did not analyse causes of asthma exacerbations and airway virus identification was not performed. Rhinovirus, the most common pathogen in asthma exacerbation^27^, upregulates neutrophilic inflammation^28^ and might contribute to the prothrombotic state reported in our study. This intriguing issue merits further investigation. The assessment of co-morbidities and medication-induced risk of asthma exacerbation were beyond the scope of our study. However, GERD and arterial hypertension (possible related to the older age of subjects who were more frequent exacerbated) were related to the increased risk of asthma exacerbation in Kaplan-Meier plots. We did not determine other potential modulators of blood coagulation and fibrinolysis, e.g. genetic polymorphisms and we cannot exclude that a prothrombotic state observed in asthma is to some extent genetically determined^29^. Statistical associations reported here may not necessarily indicate cause-effect relationships. Finally, clinical relevance of prothrombotic alterations in asthmatics in terms of thromboembolic risk and their molecular mechanisms remains to be established.

### Conclusions

Our study demonstrates that impaired fibrinolysis together with lower levels of α_2_-macroglobulin indicate an increased risk of asthma exacerbations. A larger study with long-term follow-up is needed to assess the effect of increased thrombin generation, impaired fibrinolysis and activated platelets on thromboembolic complications in asthmatics.

## Methods

### Patients and Controls

In 164 white, adult patients with clinically stable asthma, we analyzed all clinical adverse events, which occurred during follow-up (median 37, range 35–42, months). This patient population, as well as inclusion and exclusion criteria to the study, have been described previously in detail^12^. Briefly, patients were recruited at the outpatient clinic in Cracow, Poland. Asthma was diagnosed based on recurrent respiratory symptoms in the past (shortness of breath, chest tightness, wheeze, and cough) and documented post bronchodilator increase in FEV_1_ of at least 200 ml and 12% from the baseline. All asthma medications at recruitment, were permitted, with the exception of omalizumab. Oral corticosteroids were also allowed, if a daily dose was equivalent to ≤10 mg of prednisolone, with unchanged doses in preceding 3 months. Exacerbation during the last 6 months before enrolment was an exclusion criterion. Atopic status was affirmed by a positive skin prick testing for at least one inhaled allergen (Allergopharma, Reinbeck, Germany). Severity of asthma was categorized according to the Global Initiative for Asthma (GINA) guidelines^1^. Intermittent asthma was defined as treated only with inhaled short-acting β_2_-agonists on demand and not receiving inhaled or systemic corticosteroids. “Mild” asthma was defined as mild persistent disease, treated with low daily dose of inhaled corticosteroids (ICS) (<250 μg of fluticasone propionate [FP] [dry powder inhaler] or equivalent). “Moderate” asthma was defined as mild persistent disease with low (combined with long-acting β_2_-agonists) or medium dose of ICS (250–500 μg of FP or equivalent). “Severe” asthma was defined as severe persistent disease with high daily dose of ICS (>500 μg of FP or equivalent)^1^. Asthmatics were compared to the 72 control subjects matched for sex, BMI, age, co-morbidities, as well as frequency of smoking^12^. We recorded all documented clinical outcomes, including death, sudden cardiac arrest, episodes of DVT, pulmonary embolism, ischemic stroke or TIA, and ACS, as well as severe asthma exacerbations during follow-up. Data on the outcomes were collected every two months during control visits at the outpatient clinic. Moreover, in case of every asthma symptoms worsening or other clinical adverse events additional visits were also possible. The diagnosis of DVT of the lower or upper limb required a positive finding of colour duplex sonography. An iliac/caval DVT was defined as abnormal duplex flow patterns typical of thrombosis or an intraluminal filling defect on contrast computed tomography or magnetic resonance venography. The diagnosis of pulmonary embolism was based on the presence of typical symptoms and positive results of high-resolution spiral computed tomography. Stroke was defined according to the WHO criteria^30^ and demonstrated by brain imaging. TIA was defined as a transient episode of neurological dysfunction caused by a focal brain, spinal cord, or retinal ischemia, lasting <24 hours. ACS was defined according to the European Society of Cardiology guidelines^31^ as a condition resulted to: ST elevation myocardial infarction, or non ST elevation myocardial infarction, or unstable angina. Severe asthma exacerbation was defined according to the American Thoracic Society/European Respiratory Society Statement^32^ as an episode characterized by a progressive increase in asthma symptoms, which required use of systemic corticosteroids (tablets, suspension, or injection), or an increase from a stable maintenance dose, at the physician’s discretion, for at least 3 days, and/or hospitalization or emergency visit because of asthma requiring systemic corticosteroids.

The study was approved by the Ethics Committee of the Jagiellonian University.

Written informed consent was obtained from all individual participants included in the study.

All procedures performed in studies involving human participants were in accordance with the ethical standards of the research committee and with the 1964 Helsinki declaration and its later amendments or comparable ethical standards.

### Laboratory investigations

All laboratory investigations were performed at baseline before follow-up. The methodology of these tests has been previously described in detail^12^. Briefly, fasting blood samples were drawn from the antecubital vein using minimal stasis. Basic laboratory tests, including lipid profile, glucose, liver enzymes, kidney function, blood cell and platelet count, fibrinogen, hsCRP, and immunoglobulin E (IgE) were assayed by routine laboratory techniques^12^.

To assess thrombin generation, the Calibrated Automated Thrombogram was used with the subsequent calculation of prothrombin conversion independent of thrombin decay assessment, as described previously^12^. In this assay 80 μl of thawed platelet poor plasma was mixed with 20 μl of a reagent containing recombinant relipidated tissue factor and phospholipids, with the final concentrations of 5 pmol/l and 4 micromol/l, respectively. The reactions were performed in microtiter wells (Thermo Electron, Denmark) after automatic addition of a fresh starting reagent containing calcium chloride (100 mmol/l) and a thrombin specific fluorogenic substrate (Z-Gly-Gly-Arg-AMC) (2.5 mmol/l) in HEPES buffer. The fluorescence intensity was recorded by the Fluoroskan Ascent® microplate fluorometer (Thermo Fisher Scientific, Vantaa, Finland) using the software program (Thrombinoscope BV, version 3.0.0.29). The maximum concentration of thrombin during the assay time is described as the “thrombin peak” and the area under the curve represents the “endogenous thrombin potential”. “Time to thrombin peak” is the time from the start of thrombin generation until the maximum thrombin value is achieved. Thrombin dynamics analysis was performed to study differences in prothrombin conversion and thrombin inactivation as previously described^12, 18^. Prothrombin conversion was expressed as the total or maximal amount of prothrombin converted, while thrombin inactivation was quantified e.g. by thrombin-antithrombin complex formation and thrombin-α_2_-macroglobulin formation.

Plasma prothrombin and antithrombin activities were measured by the ACL TOP 500 CTS analyzer (Instrumentation Laboratory, Bedford, MA, USA). α_2_-macroglobulin, IL-6, TNFα, and platelet activation markers: PF4 and P-selectin were assessed using commercially available immunoenzymatic assays (all, R&D Systems, Minneapolis, MN, USA).

Plasminogen and antiplasmin activities, both describing fibrinolytic capacity, were analyzed by chromogenic assays (STA Stachrom plasminogen and STA Stachrom α_2_-antiplasmin, Diagnostica Stago, Asnieres, France), while PAI-1 by immunoenzymatic test (American Diagnostica, Stamford, CT, USA).

Methodology describing measurement of Clot Lysis Time (CLT), a global test of plasma fibrinolytic potential, was also described in our previous publication^12^. Briefly, in this assay plasma fibrin formation was initiated by 0.6 pmol/l human TF (Innovin, Dade Behring, Liederbach, Germany) in the presence of 15 mmol/l calcium chloride and 12 μmol/l phospholipid vesicles (Phospholipid-TGT, Rossix, Mölndal, Sweden), together with proteolysis induced by 60 ng/ml human recombinant tissue-type plasminogen activator (Boehringer Ingelheim, Ingelheim am Rhein, Germany). The mixture was transferred to a microtitre plate and fibrin optical density (OD) values were kinetically recorded (wavelength 450 nm, at 37 °C, 15 seconds per interval), using a Tecan Sunrise Instrument (Tecan, Groeding, Austria). CLT was determined as the time needed for the 50% reduction of the maximum OD value.

### Statistical Analysis

Continuous variables, all non-normal distributed (verified by the Shapiro-Wilk test), were given as median with 95% confidence interval (95% CI) and were compared by the Mann-Whitney U-test. Categorical variables were compared using χ^2^ test. To adjust for confounders: age, age of asthma onset, BMI, sex, asthma severity score according to GINA (or FEV_1_ in second analysis), systemic corticosteroid therapy at baseline and co-morbidities: hypertension and GERD, all non-normal distributed data were log-transformed and a one-way analysis of covariance (ANCOVA) was performed, which resulted in an overall p-value.

Multiple logistic regression model with Hosmer-Lemeshow test, as well as relative risk ratios (RRs) were calculated for assessment of asthma exacerbation risk with 95% CI, depending on clinical and laboratory variables. The cut-off points of numeric variables for both these analyses were calculated based on receiver operating characteristic (ROC) curves. The plots of Kaplan-Meier estimators with Cox-Mantel test were used to verify the differences in proportions of patients with and without asthma exacerbation depending on sex, age [cut-off value of 50 years], BMI [<25 kg/m^2^ and ≥25 kg/m^2^], and co-morbidities, including arterial hypertension, diabetes mellitus, GERD, and CHD, as well as the medications used. A Cox proportional hazards simple and multiple regression models, using age, age of asthma onset, sex, BMI, asthma severity (GINA), systemic corticosteroid therapy at baseline, as well as hypertension and GERD as covariates, were used to verify laboratory variables as potential independent predictors of future asthma exacerbations (first, second and all together) during follow-up.

P-values < 0.05 were considered statistically significant. Analysis was performed with the STATISTICA 12.5 software package (StatSoft, Inc, Tulsa, OK, USA).

The present study was powered to have >90% chance to demonstrate relations of clinical and laboratory variables with asthma exacerbations, using a p-value of 0.05. In order to demonstrate 12.3 or greater percent of subjects with severe exacerbations per year, the minimum number of asthmatics was 67, based on the published data^33^. However, this study was underpowered to reliable analyze the risk of thrombo-embolic events. Based on the published data on thrombotic outcomes, at least 1245 asthma subjects were required^8, 9^.

### Data Availability

The datasets analysed during the current study are available from the corresponding author on reasonable request after.
